# Distinguishing Computer-Generated Graphics from Natural Images Based on Sensor Pattern Noise and Deep Learning

**DOI:** 10.3390/s18041296

**Published:** 2018-04-23

**Authors:** Ye Yao, Weitong Hu, Wei Zhang, Ting Wu, Yun-Qing Shi

**Affiliations:** 1 School of CyberSpace, Hangzhou Dianzi University, Hangzhou 310018, China; yyaoprivate@gmail.com (Y.Y.); hwt@hdu.edu.cn (W.H.); 2 Shenzhen Key Laboratory of Media Security, Shenzhen University, Shenzhen 518060, China; 3 School of Computer Science and Technology, Hangzhou Dianzi University, Hangzhou 310018, China; 4 Department of Electrical and Computer Engineering, New Jersey Institute of Technology, Newark, NJ 07102, USA; shi@njit.edu

**Keywords:** computer-generated graphics, natural images, convolutional neural network, image forensics, sensor pattern noise

## Abstract

Computer-generated graphics (CGs) are images generated by computer software. The rapid development of computer graphics technologies has made it easier to generate photorealistic computer graphics, and these graphics are quite difficult to distinguish from natural images (NIs) with the naked eye. In this paper, we propose a method based on sensor pattern noise (SPN) and deep learning to distinguish CGs from NIs. Before being fed into our convolutional neural network (CNN)-based model, these images—CGs and NIs—are clipped into image patches. Furthermore, three high-pass filters (HPFs) are used to remove low-frequency signals, which represent the image content. These filters are also used to reveal the residual signal as well as SPN introduced by the digital camera device. Different from the traditional methods of distinguishing CGs from NIs, the proposed method utilizes a five-layer CNN to classify the input image patches. Based on the classification results of the image patches, we deploy a majority vote scheme to obtain the classification results for the full-size images. The experiments have demonstrated that (1) the proposed method with three HPFs can achieve better results than that with only one HPF or no HPF and that (2) the proposed method with three HPFs achieves 100% accuracy, although the NIs undergo a JPEG compression with a quality factor of 75.

## 1. Introduction

**Figure 1:**
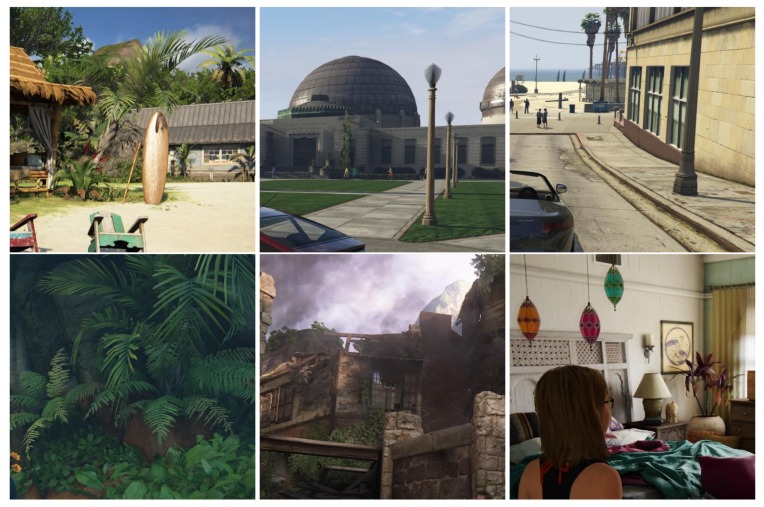
Some examples of computer graphics (CGs) from a dataset in [3].

Computer-generated graphics (CGs) are images generated by computer software. In recent years, with the aid of computer software, it is easier to generate photorealistic computer graphics (PRCGs), which are quite difficult to distinguish from natural images (NIs) with the naked eye. Some examples of computer graphics are shown in Figure 1. Although these rendering software suites help us to conveniently create images and animation, it could also bring serious security issues to the public if PRCGs were used in fields such as justice and journalism [1]. Therefore, as an essential topic in the domain of digital image forensics [2], distinguishing CGs from NIs has attracted increasing attention in the past decade.

Several algorithms have recently been proposed to distinguish CGs from NIs. Xiaofen Wang et al. [4] present a customized statistical model based on the homomorphic filter and use support vector machines (SVMs) as a classifier to discriminate photorealistic computer graphics (PRCGs) from NIs. Zhaohong Li et al. [5] present a multiresolution approach to distinguishing CGs from NIs based on local binary patterns (LBPs) features and an SVM classifier. Jinwei Wang et al. [6] present a classification method based on the first four statistical features extracted from the quaternion wavelet transform (QWT) domain. Fei Peng et al. [7] present a method to extract 24 dimensions of features based on multi-fractal and regression analysis for the discrimination of CGs and NIs. However, all of these methods have depended on handcrafted algorithm to extract features. That is to say, these features are designed by researchers, rather than learned from CGs and NIs by a machine-learning algorithm.

Deep learning has been used in many new fields and has achieved great success in recent years. Deep neural networks such as the convolutional neural network (CNN) have the capacity to automatically obtain high-dimensional features and reduce its dimensionality efficiently [8]. Some researchers have begun to utilize deep learning to solve problems in the domain of image forensics, such as image manipulation detection [9], camera model identification [10,11], steganalysis [12,13], image copy-move forgery detection [14], and so on.

In this paper, we propose a method based on sensor pattern noise (SPN) and deep learning to distinguish CGs from NIs. Different from the traditional methods of distinguishing CGs from NIs, the proposed approach utilizes a five-layer convolutional neural network (CNN) to make a classification for the input images. Before being fed into the CNN-based model, these images—including the CGs and NIs—are clipped into image patches. Three high-pass filters (HPFs) are used to remove low-frequency signals, which represent image content. These filters are also used to reveal the residual signal as well as SPN introduced by the digital camera device. The experimental results have shown that the proposed method with three HPFs can achieve 100% accuracy, although the NIs undergo a JPEG compression with a quality factor of 75.

## 2. Related Works

There are several studies related to deep learning as well as SPN used for forensics.

### 2.1. Sensor Pattern Noise Used for Forensics

Different digital cameras introduce different pattern noise to their output digital images. One of the pattern noise sources is due to the imperfection of CCD or CMOS sensors. This has been named “sensor pattern noise” (SPN) and is used as a fingerprint to characterize an individual camera. In particular, SPN has been used in image forgery detection [15] and source camera identification [16].

Villalba et al. [17] presented a method for video source acquisition identification based on SPN extraction from video key frames. Photo response non-uniformity (PRNU) is the primary part of the SPN in an image. In [17], the PRNU is used to calculate the SPN and characterize the fingerprints into feature vectors. The feature vectors are then extracted from the video key frames and trained by an SVM-based classifier.

### 2.2. Methods Based on Deep Learning

Gando et al. [18] presented a deep learning method based on a fine-tuned deep convolutional neural network. This method can automatically distinguish illustrations from photographs. The illustrations–photographs dataset and the illustrations–cosplays dataset was constructed for model evaluation. The fine-tuned CNN model on the illustrations–photographs dataset achieved a 96.8% accuracy. The model with five convolution layers obtained a 93.0% accuracy on the illustrations–cosplays dataset. It outperforms other models, including custom CNN-based models trained from scratch and traditional models using handcrafted features.

Rahmouni et al. [3] presented a custom pooling layer to extract statistical features and a CNN framework to distinguish CGs from real photographic images. A weighted voting scheme was used to aggregate the local estimates of class probabilities and predict the label of the whole picture. The CGs used in [3] were downloaded from the Level-Design Reference Database [19]. The NIs were taken from the RAISE dataset [20]. The best accuracy in [3] was 93.2%, obtained by the proposed Stats-2L model.

Rezende et al. [21] presented a deep convolutional neural network model for CG detection based on ResNet-50 and transfer learning. It is able to distinguish CGs from photo images without any pre-processing or hand-crafted feature extraction. The trained model obtained an accuracy higher than 94% on a public dataset comprising 9700 images.

In this paper, we propose a CNN-based method of distinguishing CGs from NIs based on SPN and deep learning. This CNN-based model consists of a five-layer convolutional neural network. It is much simpler than Rezende et al.’s method [21], which is based on ResNet-50 and transfer learning.

## 3. The Proposed Method

The proposed method consists of two primary steps: image preprocessing and CNN-based model training. In the first step, the input images—the CGs and the NIs—are clipped into image patches, then three HPFs are applied to the image patches. These filtered image patches constitute positive and negative training samples. In the second step, the filtered image patches are fed to the proposed CNN-based model for training. The proposed CNN-based model is a five-layer CNN. In this section, we introduce the steps of our method in detail.

### 3.1. Image Preprocessing

#### 3.1.1. Clipping into Image Patches

The NIs taken by cameras and the CGs generated by software often have a large resolution. Due to hardware memory limitations, we needed to clip these full-size images into smaller image patches before they were fed into our neural network for training. This is also a data augmentation strategy in deep learning approaches to computer vision [8]. Data augmentation [22] helps to increase the amount of training samples used for deep learning training and improve the generalization capability of the trained model. Therefore, we clipped all of the full-size images into image patches. The resolution of each image patch is 650 × 650. We chose this size as a trade-off between processing time and computational limitations.

All clipping is a label-preserving operation. That is to say, we prepared the positive samples by drawing image patches from the full-size NIs. In a similar way, we obtained negative samples from the full-size CGs. However, NIs taken by cameras usually have a larger resolution than CGs. If we want the amount of negative samples and the amount of positive samples to be approximately equivalent, we need to clip image patches from CGs more so than we do from NIs. In light of this, we set the stride size for NIs to the width of the image patches (i.e., 650). After analyzing the amount of image patches, we set the stride size for CGs to a smaller value (i.e., 65).

#### 3.1.2. Filtering with High-Pass Filters

Since the NIs and the CGs are created from different pipelines, distinct differences between them are expected. As is known, SPN has been used to identify source cameras of NIs and has obtained excellent performance [10,11,16]. However, there is no SPN in CGs. Based on this idea, we propose our method to discriminate CGs from NIs.

Fridrich et al. [23] designed several HPFs for the steganalysis of digital images. As mentioned, these filters have the ability to obtain image residuals and suppress the value of the low-frequency component, which represents the image content. Qian et al. [24] proposed a customized convolutional neural network for steganalysis. This customized deep learning approach starts with a predefined HPF. This predefined HPF was proposed as an image residual extraction model of a SQUARE 5 × 5 filter in [23]. Furthermore, this image residual extraction model has been applied to deep-learning-based camera model identification [11] as well as to deep-learning-based video forgery detection [8] and has obtained perfect performance.

In this paper, we utilize several HPFs in our method to reveal the SPN and reduce the impact of the image content. The SPN is included in the image residuals. These predefined HPFs are employed to make a convolution operation with the image patches to obtain image residuals. Furthermore, in order to reduce the computational complexity, the image patches are first converted to grayscale. The predefined HPFs are applied to the grayscale image patches, and the image residuals of the image patches are then piped into the proposed convolutional neural network.

**Figure 2:**
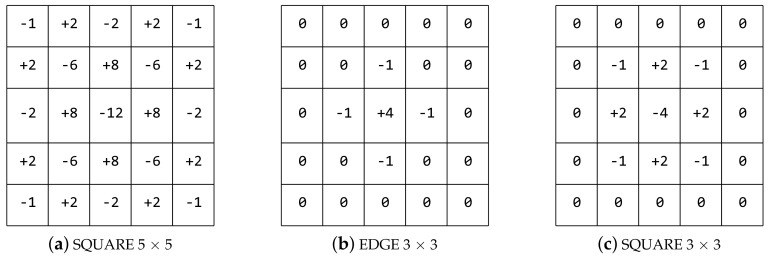
Three high-pass filter (HPFs) used in the proposed method. (**a**) the high-pass filter of SQUARE 5 × 5, (**b**) the high-pass filter of EDGE 3 × 3, (**c**) the high-pass filter of SQUARE 3 × 3.

The proposed HPFs are shown in Figure 2. The SQUARE 5 × 5 and SQUARE 3 × 3 were proposed as image residuals extraction models in [23]. The EDGE 3 × 3 was designed by us according to the different structure of the other filters in [23]. In order to ensure that the three filters have the same size, the elements in the bounding boxes of the SQUARE 3 × 3 and the EDGE 3 × 3 are set to zero.

In our method, we define three types of combinations for the three HPFs. The combination of the *High_Pass_Filter × 3* consists of all three proposed filters, i.e., the SQUARE 5 × 5, the EDGE 3 × 3, and the SQUARE 3 × 3. The combination of the *High_Pass_Filter × 1* only contains the SQUARE 5 × 5 filter. The combination of the *High_Pass_Filter × 0* utilizes an average pooling layer instead of the HPF layer, where × 0 means that there is no high-pass filter. We select the combination of the *High_Pass_Filter × 3* for our proposed method. The other combinations are considered for comparison.

### 3.2. CNN-Based Model Training

**Figure 3:**
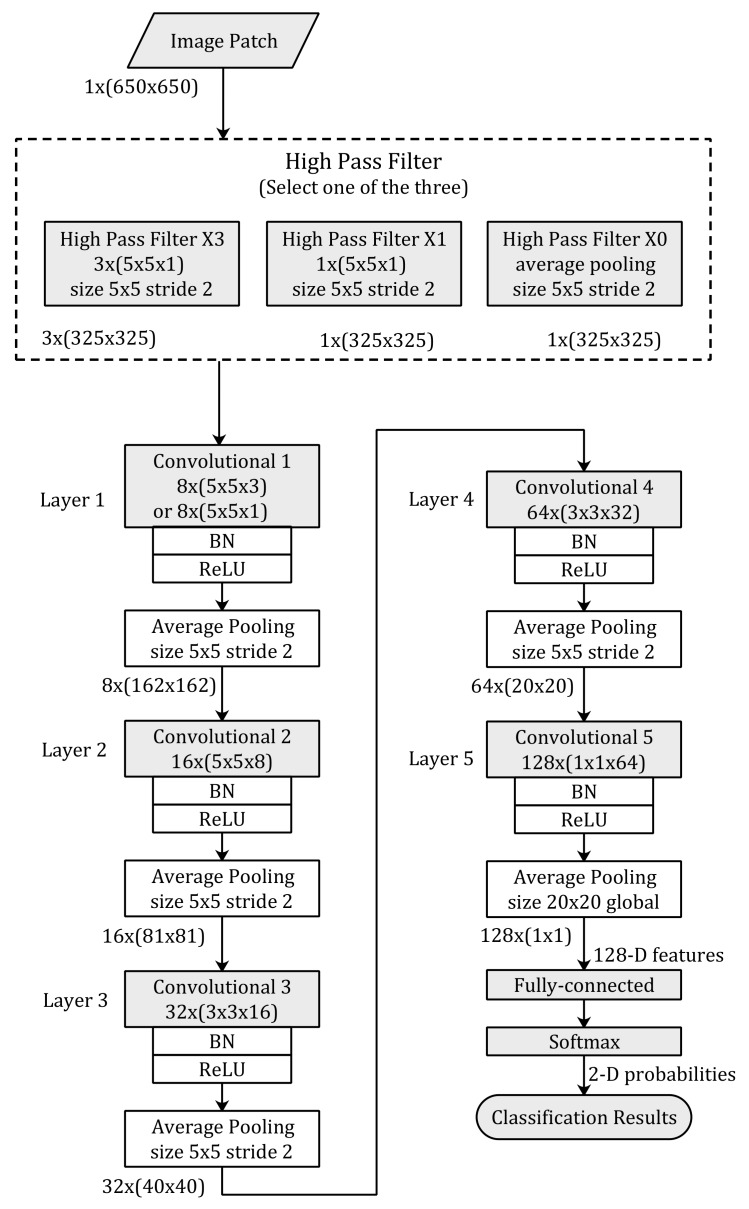
The proposed convolutional neural network architecture. Names and parameters of each layer are displayed in the boxes. Kernel sizes in each convolution layer are shown as *number_of_kernels* × (*width* × *height* × *number_of_input*). Sizes of feature maps between consecutive layers are shown as *number_of_feature_maps* × (*width* × *height*). Padding is used in each convolutional layer to keep the shape of image patches. BN: batch normalization; ReLUs: rectified linear units.

The proposed convolutional neural network architecture is illustrated in Figure 3. The image patches of the input for the proposed neural network are image blocks clipped from the full-size NIs or CGs with a resolution of 1 × (650 × 650), where 1 represents the channel number of gray-scale, and 650 figures represent the width and height, severally.

There is an HPF layer at the top of the proposed CNN-based model. This filter layer consists of three combinations of HPFs. We need to select one type of the three combinations for the deep learning training. According to the combination used by the method, the number of feature maps outputted by the filter layer is different. If the *High_Pass_Filter × 3* is used, there will be three feature maps with a size of 325 × 325 outputted by the HPF layer. Otherwise, there will only be one feature map of a size of 325 × 325 outputted by the HPF layer.

The proposed CNN architecture consists of five convolutional layers. Each convolutional layer is followed by a batch normalization (BN) [25] layer, a rectified linear units (ReLUs) [26] layer, and an average pooling layer. For the selection of the number of layer, the activation function, and a BN layer, we mainly refer to the existing works in the field of deep-learning-based forensics [12]. At the bottom of the proposed model, a fully connected layer and a softmax layer are utilized to transform the 128 dimensional feature vectors to classification probabilities of the image patches.

The kernel sizes of the convolution layers in the proposed CNN-based model are 5 × 5, 5 × 5, 3 × 3, 3 × 3, and 1 × 1, respectively. The amounts of the feature maps outputted by each layer are 8, 16, 32, 64, and 128, respectively, and the size of feature maps are 325 × 325, 162 × 162, 81 × 81, 40 × 40, and 20 × 20, respectively. The kernel size of the average pooling in each layer is 5 × 5 and the stride size is 2. Note that the last average pooling layer has a global kernel size of 20 × 20.

## 4. Experiments

### 4.1. Dataset

We compared our deep learning approach with the state-of-the-art methods in [3]. The dataset used in this paper is the same as the dataset in [3]. It consists of 1800 CGs and 1800 NIs. The CGs were downloaded from the Level-Design Reference Database [19], which contains more than 60,000 screenshots of video games. The game information was removed by cropping the images to a resolution of 1280 × 650. The preprocessed images can be downloaded from the link on Github [27]. Some CG samples are shown in Figure 1. The NIs are taken from the RAISE dataset [20]. The resolution of these NIs ranges from 3008 × 2000 to 4900 × 3200. All of these NIs were downloaded in RAW format and converted to JPEG with a quality factor of 95.

In our experiment, 900 NIs and 900 CGs were randomly selected from the dataset for training, 800 NIs and 800 CGs were set aside for testing, and 100 NIs and 100 CGs set aside for validation. Then, all of these full-size images were clipped into image patches with a size of 650 × 650. The number of image patches we obtained for training was about 44,000.

### 4.2. Experiment Setup

We implemented the proposed convolution neural network based on the Caffe framework [28]. All of the experiments were conducted on a GeForce GTX 1080ti GPU. The stochastic gradient descent algorithm was used to optimize the proposed CNN-based model. The initial learning rate was set to 0.001. The learning rate update policy was set to inv with a gamma value of 0.0001 and a power value of 0.75. The parameters of momentum and weight_decay were set to 0.9 and 0.0005, respectively. The batch size of training was set to 64. Namely, 64 image patches were fed to the CNN-based model for each iteration. After 80 epochs of iteration, the trained CNN-based model was obtained for testing.

In order to get the performance of the proposed CNN-based model, we applied the trained model to the testing dataset. All of the full-size images in the testing dataset needed to be preprocessed. The preprocessing for the testing images was similar to the preprocessing of the images in the training. After preprocessing, the testing images were clipped into image patches. These image patches were then fed to the trained CNN-based model, and the prediction results for the image patches were obtained. Based on the prediction results of the image patches, we deployed a majority vote scheme to obtain the classification results for the full-size images.

### 4.3. Experimental Results

#### 4.3.1. Different Numbers of High-Pass Filters

**Figure 4:**
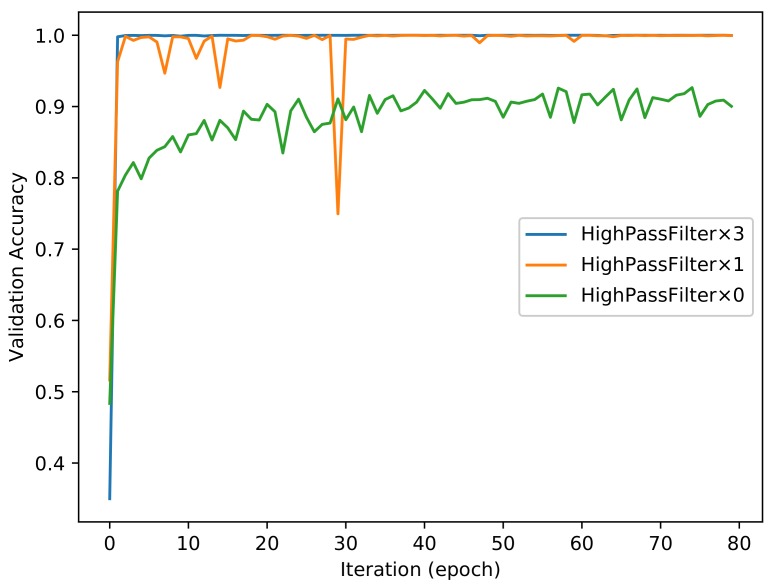
Validation performance of the proposed method.

**Figure 5:**
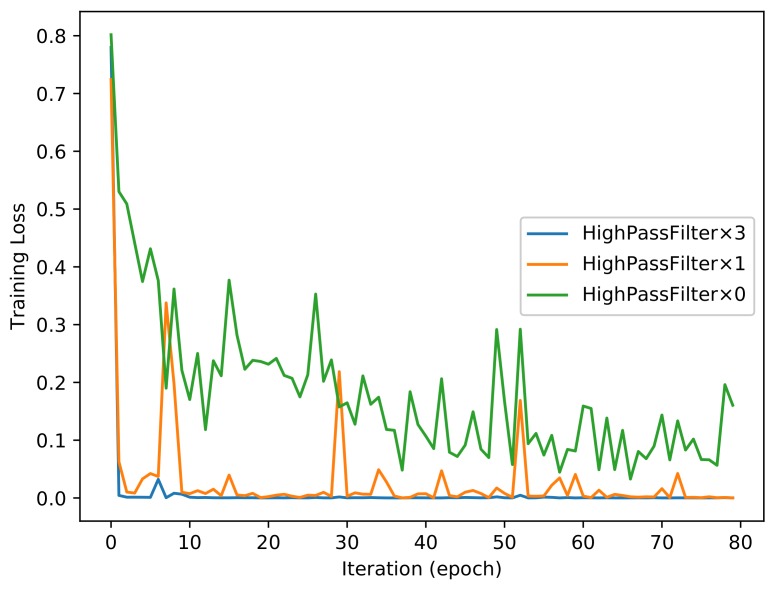
Training loss of the proposed method.

As shown in Figure 3, the proposed convolution neural network has three combinations for the HPF layer. Each of the combinations has different numbers of HPFs. We trained all of these combinations for 80 epoch iterations and obtained two trained models for each of the combinations. In other words, we obtained a model of 50 epochs and a model of 80 epochs for the combination of *High_Pass_Filter × 3* after training the proposed network for 80 epoch iterations. We also obtained the same number of models for the other two combinations. Figure 4 and Figure 5 show the evolutions of training loss and validation accuracy in the procedure of iteration. The validation accuracy is shown in Figure 4, and the training loss is shown in Figure 5. It is observed that the proposed method with *High_Pass_Filter × 3* converges much faster than the others and achieves much higher prediction accuracy.

To evaluate the classification performance of the proposed method with different numbers of HPFs, we tested these models obtained in the training procedure on the testing dataset. The classification accuracy is shown in Table 1. Note that the size of the image patches in the method of Rahmouni et al. in [3] is 100 × 100. In our experiments, we set the size of the image patches to 650 × 650 to meet the requirement of our neural network architecture. A majority vote scheme was applied to the testing results of the image patches to obtain the classification results for the full-size images.

Our method with the HPF outperformed the state-of-the-art method of Rahmouni et al. in [3]. Furthermore, the proposed method with *High_Pass_Filter × 3* outperformed the other filter combinations and obtained the best performance. The classification accuracy for the full-size images reached as high as 100%. These experimental results demonstrate the effectiveness of the HPF in the preprocessing procedure for our proposed deep learning approach.

#### 4.3.2. Different Quality Factors of Natural Images

We also evaluated the robustness of our proposed method in relation to different quality factors. In this experiment, 2000 NIs in RAW format were downloaded from the RAISE-2k dataset [20]. We randomly selected 1800 NIs for our robustness experiment. These RAW images were converted to JPEG format with quality factors of 95, 85, and 75, respectively. We then obtained three sub-datasets with different quality factors of NIs for our experiment. Each of the sub-datasets were then divided into training (50%), testing (40%), and validation (10%) to form the datasets for the robustness experiment of quality factors. Note that the CGs in this experiment remained untouched. These CGs were compressed with a reasonable quality factor when the author collected this dataset.

**Figure 6:**
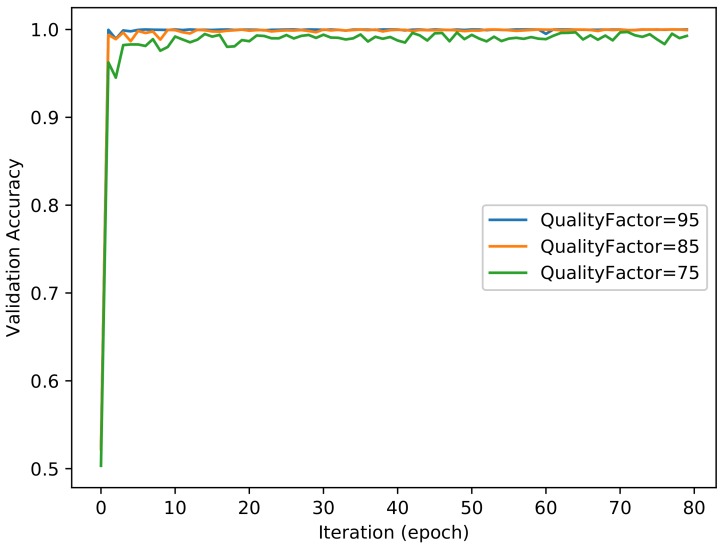
Validation performance of the proposed method.

**Figure 7:**
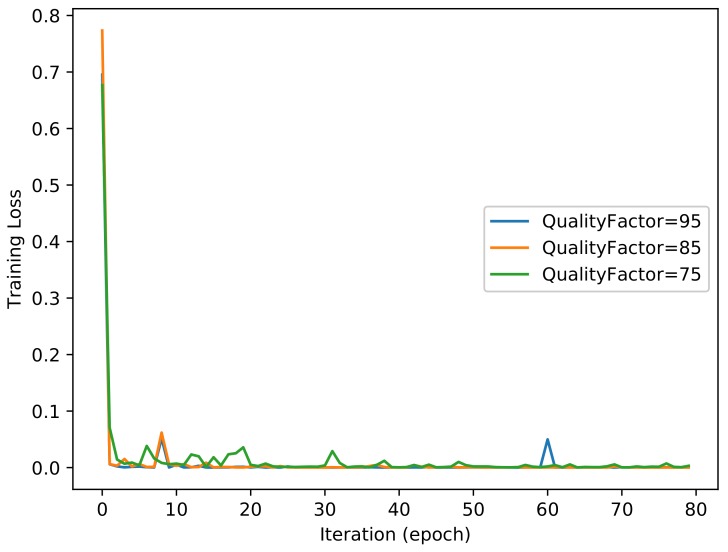
Training loss of the proposed method.

For the filter layer, we utilized *High_Pass_Filter × 3* to achieve the best performance in this experiment. Figure 6 and Figure 7 show the evolutions of training loss and validation accuracy in the iteration procedure. The validation accuracy is shown in Figure 6, and the training loss is shown in Figure 7. The classification accuracy for different quality factors of NIs is shown in Table 2. It was observed that the proposed method with *High_Pass_Filter × 3* achieves a perfect performance. Although the compression with different quality factors has an impact on the classification accuracy of image patches, due to the majority vote scheme used for the full-size images, all of the classification accuracies for different quality factors of the NIs are 100%.

## 5. Conclusions

In this paper, we develop an approach to distinguish CGs from NIs based on SPN and a convolutional neural network. The experimental results show that the proposed method outperforms the method in [3] on the same dataset. Currently, there are several CG datasets [5,7] for forensics research. However, many images in these datasets are smaller than 650 pixels in width or height. This does not meet the size requirement of the proposed convolutional neural network. In the future, we will focus on the improvement of our CNN-based model for smaller images and try to apply a trained CNN-based model to discriminate CGs from other existing datasets, namely one model for all datasets. Furthermore, some [29] have proposed the task of classifying realistic vs. unrealistic photos by means of learning the perception of visual realism from automatically generated composite images. Others [30] have suggested taking the opposite route: using 3D graphics to generate a dataset for model training of specific real objects. These ideas would be worth exploring in future works.

## Figures and Tables

**Table 1:** Classification accuracy with different numbers of HPFs.

	Image Patches		Full-Size Images	
	Model of 50 Epochs	Model of 80 Epochs	Model of 50 Epochs	Model of 80 Epochs
the proposed HPF × 3	99.98%	99.95%	100%	100%
the proposed HPF × 1	99.87%	99.77%	100%	99.83%
the proposed HPF × 0	88.28%	87.77%	93.37%	93.12%
Rahmouni et al. [3]	84.8% ^1^ 94.4% ^2^		93.2%	

^1^ The size of the image patches was 100 × 100, which is different from the proposed methods. ^2^ The size of the image patches was 650 × 650. A weighted voting scheme was used.

**Table 2:** Classification accuracy for different quality factors of natural images (NIs).

	Image Patches		Full-Size Images	
	Model of 50 Epochs	Model of 80 Epochs	Model of 50 Epochs	Model of 80 Epochs
*QualityFactor* = 75	99.52%	99.71%	100%	100%
*QualityFactor* = 85	99.95%	99.98%	100%	100%
*QualityFactor* = 95	99.99%	99.99%	100%	100%
